# 

**Published:** 1892-12-31

**Authors:** 


### CONTENTS.

| | PAGE |
|---|---|
| "This Above All" | 211 |
| Passing Topics.—Foul Language in Public Places | 212 |
| Diet in Disease. By Mrs. Ernest Hart. XI. — Invalid Foods | 213 |
| Editor's Letter-Box. — Cheyne Hospital for Sick and Incurable Children, Cheyne Walk, Chelsea | 214 |
| Medical History from the Earliest Times. By E. T. Withington, M.A., M.B.Oxon. XXXVII.—The Ladies of Salerno | 215 |
| Everybody's Page | 216 |
| Annotations.—"Ring Out the Old"—"Nor Friends nor Money"—Trained Almoners—M. Pasteur's Seventieth Birthday | 217 |
| Notes and News | 218 |
| The Hospital Clinic— | |
| The London Hospital.—The Treatment of Injuries to the Hand | 219 |
| The Bradford Infirmary.—The Treatment of Slighter Forms of In-Knee | 220 |
| Royal Free Hospital.—The Treatment of Some Forms of Heart Disease | 221 |
| New Drugs, Appliances, and Things Medical.—An Antiseptic Hypodermic Syringe | 222 |
| Christmas Fare for the Children | 223 |
| The People's Needs.—The Social Horizon | 225 |
| The Student of Medicine.—Death of a Bristol Student—Appointments—Vacancies | |

### EXTRA SUPPLEMENT.—THE NURSING MIRROR.

| | |
|---|---|
| En Passant.—Arbroath District Nursing Association—Cotswold Benefit Nursing Association—Short Items—Edinburgh Royal Infirmary—St. Luke's Home, Vancouver—London Diocesan Council for Preventive Work—Manchester and Salford Sick Poor and Private Nursing Institution—The Scottish Branch of the Queen Victoria Jubilee Institute | ci |
| The Development of Children by Gymnastics.—II. | cii |
| Nursing Homes.—VI. Royal Free Hospital | ciii |
| Novelties for Nurses | ciii |
| Having or Giving? | civ |
| Wants and Workers | civ |
| Everybody's Opinion.—Nurses' Fees—The Latest Hospital Authority | civ |
| Presentations | civ |
| Appointments—Minor Appointments | cv |
| Notes and Queries | cv |
| For Reading to the Sick.—The New Year | cv |
| A Nursing Worthy.—Mrs. Wardroper | cvi |

